# A systematic review and meta-analysis for Chinese herbal medicine Duhuo Jisheng decoction in treatment of lumbar disc herniation: A protocol for a systematic review

**DOI:** 10.1097/MD.0000000000019310

**Published:** 2020-02-28

**Authors:** Kai Sun, Fasen Huang, Baoyu Qi, He Yin, Bin Tang, Bowen Yang, Lin Chen, Minghui Zhuang, Xu Wei, Liguo Zhu

**Affiliations:** a Department of Spine, Wangjing Hospital of China Academy of Chinese Medical Sciences; b The Third Affiliated Hospital of Beijing University of Chinese Medicine; c Office of Academic Research, Wangjing Hospital of China Academy of Chinese Medical Sciences, Beijing, China.

**Keywords:** duhuo jisheng decoction, effectiveness and safety, lumbar disc herniation, randomized controlled trials, systematic review

## Abstract

**Background:**

Lumbar disc herniation (LDH) is 1 of the most common diseases in orthopedics, which seriously affects people's daily life and brings a heavy burden on society and families. Chinese herbal medicine has been used in clinical practice for a long time and Duhuo Jisheng Decoction (DHJSD) is believed to help alleviate the symptoms of LDH. This systematic review aims to collect evidences from randomized clinical trials and evaluate the efficacy of DHJSD on LDH in order to provide a reference for clinicians and researchers.

**Methods:**

We will comprehensively search the 8 electronic databases until December 2019 to identify related randomized controlled trials, including 4 foreign databases (PubMed, MEDLINE, EMBASE, Cochrane Library) and 4 Chinese databases (China National Knowledge Infrastructure Database, VIP Database, Wanfang Database and China Biology Medicine disc). The data of the World Health Organization International Clinical Trial Registry Platform and the Chinese Clinical Trial Registry also will be searched. The primary outcomes are Japanese Orthopaedic Association scores and visual analog scale scores. The risk of bias will be assessed using the Cochrane Collaboration tool. RevMan (V.5.3) software will be used for meta-analysis.

**Results:**

This study will report the results of DHJSD for the treatment of LDH from the literature screening, the basic information of the included studies, the risk of bias of the included studies, treatment effects, safety, and so on.

**Conclusion:**

This systematic review will evaluate the effectiveness and safety of DHJSD for the treatment of LDH and provide the latest evidence for its clinical application.

**Ethics and dissemination:**

This is a literature-based study, therefore it does not require ethical approval.

**PROSPERO registration number:**

CRD42019147302.

## 1 Introduction

Lumbar disc herniation (LDH) is 1 of the most common diseases in orthopedics. It mainly manifests as low back pain and sciatica caused by nerve compression, and may also include radiating pain, motor weakness and sensory disturbances.^[1–4]^ Studies have shown that the highest prevalence was between ages 45 and 64, with more male patients than female patients. The incidence of L4/5 and L5/S1 is higher than other sections, even more than 90%.^[1,3,5]^ With the aging of the social population and changes in people's lifestyles, the incidence of LDH has increased significantly. LDH is characterized by a high disability rate, which has brought a heavy burden on society and families.^[6,7]^

At present, the methods of treatment of LDH include surgical therapy and nonsurgical therapy. Surgical treatment mainly adopts methods such as fixation, decompression and fusion (anterior lumbar interbody fusion, posterior lumbar interbody fusion, open transforaminal lumbar interbody fusion and so on) to reduce the clinical symptoms of patients, but there are problems such as wound infection, complications, and high postoperative recurrence rate.^[8–10]^ Non-surgical treatments include traction, manipulation, acupuncture, physiotherapy and medicines (Chinese medicine and non-steroidal anti-inflammatory drugs) and so on. Many randomized controlled trials (RCTs) or systematic reviews have proven that these methods alone or comprehensively have a certain effect for the treatment of LDH.^[11–14]^

In recent years, more and more people have paid attention to traditional Chinese medicine (TCM) therapy. The ancient classic prescriptions represented by Duhuo Jisheng Decoction (DHJSD) have been widely used in the treatment of LDH.^[15,16]^ DHJSD is recorded in the book “Bei Ji Qian Jin Yao fang” written by Sun Simiao of Tang Dynasty. It consists of 15 commonly used Chinese herbs, such as Duhuo (Radix Angelicae Biseratae), Sangjisheng (Herba Taxilli), Duzhong (Eucommiae Cortex), Niuxi (Achyranthis Bidentatae Radix), Xixin (Asari Radix Et Rhizoma), Qinjiao (Gentiana Macrophylla Pall), Fuling (Poria Cocos [Schw.] Wolf.), Rougui (Cinnanmomi Cortex), Fangfeng (Saposhnikoviae Radix), Chuanxiong (Chuanxiong Rhizoma), Renshen (Panax Ginseng C. A. Mey.), Danggui (Angelicae Sinensis Radix), Baishao (Paeoniae Radix Alba), Shudihuang (Rehmanniae Radix Praeparata), and Gancao (licorice). According to the theory of TCM, it has the effects of eliminating rheumatism, analgesics, nourishing liver and kidney, nourishing Qi and blood, and channeling meridians.^[17,18]^ Of course, it is also used to treat other orthopedic diseases, such as knee arthritis and osteoporosis.^[19,20]^ Although there has been a systematic review on the treatment of LDH with DHJSD before,^[21]^ the latest standardized systematic reviews need to be carried out with the addition of a large number of clinical trials.^[15,16]^ Therefore, the purpose of this study is to summarize the results of current RCTs, analyze the effectiveness and safety of DHJSD for the treatment of LDH, and provide evidence-based medicine for its better application.

## 2 Methods

### 2.1 Study registration

This systematic review protocol has been registered on the Prospective Register of Systematic Reviews (PROSPERO). The approved registration number is CRD42019147302. The details of the protocol for this systematic review can be accessed at https://www.crd.york.ac.uk/PROSPERO/#recordDetails.

### 2.2 Ethics and dissemination

This is a literature-based study and therefore it does not require ethical approval.

### 2.3 Inclusion criteria

#### 2.3.1 Study design

RCTs of all eligible published DHJSD for the treatment of LDH. Language is limited in Chinese and English. Case reports, case series, non-RCTs, quasi-RCTs, animal experiments, cell experiments and other studies will be not included.

#### 2.3.2 Participants

Patients need to be clearly diagnosed with LDH and choose conservative treatment. The region, sex, age, nation, and duration of disease are not limited.

#### 2.3.3 Types of interventions

The patients in the experimental group were treated with DHJSD alone, or treated with DHJSD on the basis of the control group. The intervention methods of the control group were conventional treatments (non-steroidal antiinflammatory drugs, nutritional nerve drugs, and so on.) or placebo. The patients of both groups required oral administration. The dosage and duration of treatment were not limited.

#### 2.3.4 Types of outcome measures

This protocol proposes to assess the effectiveness of DHJSD for LDH by using the primary outcomes of Japanese Orthopaedic Association scores, visual analog scale scores. We also use the following outcomes as secondary outcome indicators: Clinical effectiveness, symptom scores, adverse events. The clinical effectiveness standard refers to the guiding principles for Clinical Trials of New Patent Chinese Medicines.^[22]^

### 2.4 Exclusion criteria

The types of excluded literatures mainly include: literatures of which the complete data cannot be obtained; literatures with incorrect data; literatures with random methods or intervention methods that are incorrect, and so on. For duplicate data, only the first 1 will be retained.

### 2.5 Search strategy and study selection

#### 2.5.1 Database resources

We will comprehensively search the following electronic databases, including 4 foreign databases (PubMed, MEDLINE, EMBASE, Cochrane Library) and 4 Chinese databases (China National Knowledge Infrastructure Database, VIP Database, Wanfang Database and China Biology Medicine disc). In addition, the data of the WHO International Clinical Trial Registry Platform and the Chinese Clinical Trial Registry also will be searched. All electronic databases will be searched without regional restrictions and the search time will start from the construction of the database to December 2019. We will not search for articles that have not been published.

#### 2.5.2 Search strategy

The search strategy will be based on Cochrane Handbook guidelines^[23]^ and it will be searched individually or in combination using the following terms: “Duhuo Jisheng”, “Duhuojisheng,”“Chinese herbal medicine,”“lumbar disc herniation,” “LDH,” “lumbosacral radiculopathy.” Each database will be searched according to the requirements. The specific search strategy will be as follows, such as the PubMed database: (Duhuo Jisheng [title/abstract]) OR Duhuojisheng [title/abstract]) OR Chinese herbal medicine (Title/Abstract) AND (LDH [title/abstract]) OR LDH [title/abstract]) OR lumbosacral radiculopathy [title/abstract]).

#### 2.5.3 Study selection

**Figure 1:**
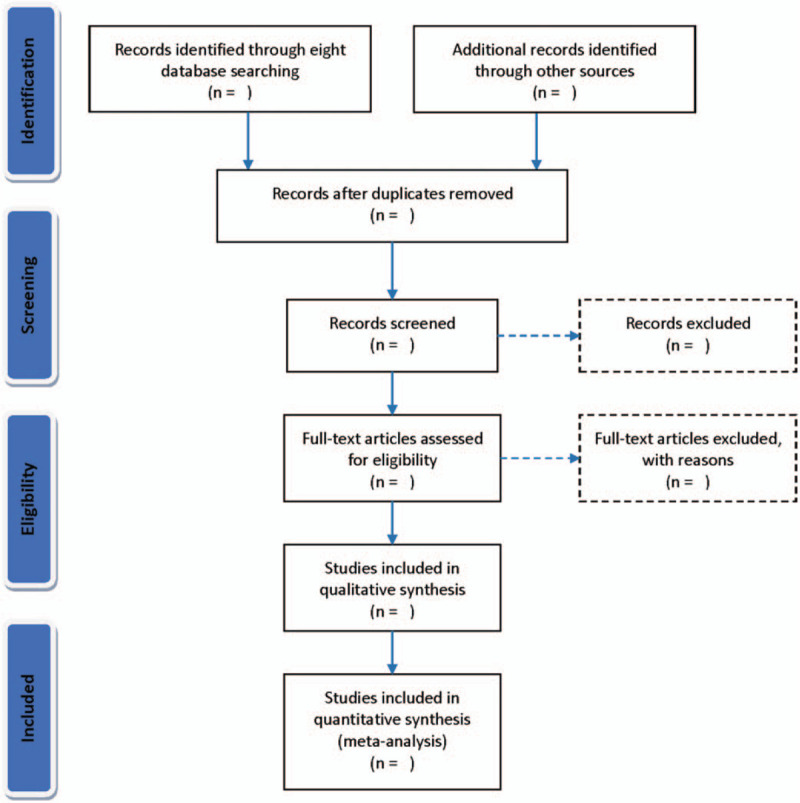
Flow diagram of study selection process.

The 2 researchers will independently search the literatures according to the formulated requirements and use Note Express (V.3.2.0) software for management. After excluding duplicate and totally irrelevant literatures, read the full text to determine the final included literatures. When 2 researchers encounter difference, we will discuss with a third researcher together. The details of study selection will be summarized by using a PRISMA flow diagram (Fig. 1).

### 2.6 Data extraction and quality assessment

#### 2.6.1 Data extraction

The data extraction will be done independently by 2 researchers who will use an uniform standard form to extract the required information from each eligible original study. It contains the following information: author, year of publication, country, age, gender, sample size, random method, blind method, follow-up information, intervention method, research results, safety, and so on. Any differences in data extraction between the 2 researchers will be resolved by a third researcher.

#### 2.6.2 Assessment of risk of bias in included studies

We will assess the risk of literature bias using the Cochrane Collaboration's tool.^[24]^ 2 researchers will independently assess methodological quality using the following 7 aspects of the RevMan software (V.5.3): Random sequence generation, Allocation concealment, Blinding of participants and personnel, Blinding of outcome assessment, Incomplete outcome data, Selective reporting, and Other bias. Each entry will be evaluated at 3 levels: low risk of bias, unclear risk of bias and high risk of bias. As before, this will be completed under the supervision of a third researcher.

### 2.7 Data analysis

#### 2.7.1 Statistical analysis

We will use RevMan software (V.5.3) provided by the Cochrane Collaboration for data analysis. In view of the characteristics of the previously extracted data, for dichotomous data, we will express the results as a risk ratio with a 95% confidence intervals. For continuous data, the mean difference will be included in the meta-analysis. When *I*^2^ ≤50%, we will choose fixed effect model analysis; when *I*^2^ > 50%, we will choose random effect model analysis. If the data cannot be analyzed quantitatively, we will perform a qualitative analysis of the data.

#### 2.7.2 Subgroup analysis

If the included studies have greater heterogeneity, we will perform subgroup analysis to explore the source of heterogeneity, which may be explored in terms of disease course, treatment course, and so on.

#### 2.7.3 Sensitivity analysis

If necessary, we will perform a sensitivity analysis of the results to check the stability of the analysis conclusions. For example, We will delete the studies that have a high risk of bias.

#### 2.7.4 Reporting bias analysis

According to the results of the study, if there are enough studies for the meta-analysis (n ≥10), we will evaluate the reporting bias through funnel plots.

## 3 Discussion

The pathogenesis of LDH is very complicated. At present, it is considered to be related to nerve compression, chemical inflammation, autoimmunity, and biomechanics.^[25,26]^ As we all know, as a common treatment method for LDH, Chinese herbal medicine has been used in clinical practice for a long time. DHJSD is 1 of the representative prescriptions.^[27,28]^ Basic research shows that it has the effects of antiinflammatory, analgesia, immune regulation, cartilage promotion, and fibrous ring repair. Liu et al^[29]^ discovered that DHJSD could inhibit the generation of proinflammatory mediators and extracellular matrix degradation through the SDF-1/CXCR4/NF-κB pathway. Similarly, Liu et al^[30]^ demonstrated that DHJSD could prevent compression-induced matrix degradation and cell apoptosis through regulating autophagy and the P38/MAPK signaling pathway in vivo and in vitro experiments. These are good results obtained in basic experiments. Unfortunately, the clinical trials of DHJSD for LDH still lack comprehensive systematic reviews and research evidences.

Therefore, we aim to summarize the currently published evidences and try to evaluate the effectiveness and safety of DHJSD in treating LDH through this systematic review. Although this study may have some limitations, we hope to further promote clinical practice and provide inspiration and reference for scientific researchers and clinicians.

## Author contributions

**Conceptualization:** Kai Sun, Fasen Huang, Baoyu Qi.

**Data curation:** He Yin, Bin Tang.

**Investigation:** Bowen Yang, Lin Chen.

**Methodology:** Xu Wei, Liguo Zhu.

**Resources:** Bowen Yang, Minghui Zhuang.

**Software:** Bin Tang, Minghui Zhuang.

**Supervision:** He Yin, Lin Chen.

**Writing – original draft:** Kai Sun, Fasen Huang, Baoyu Qi.

**Writing – review and editing:** Xu Wei, Liguo Zhu.
